# Nectins and Nectin-like Molecules in Colorectal Cancer: Role in Diagnostics, Prognostic Values, and Emerging Treatment Options: A Literature Review

**DOI:** 10.3390/diagnostics12123076

**Published:** 2022-12-07

**Authors:** Jakub Kobecki, Paweł Gajdzis, Grzegorz Mazur, Mariusz Chabowski

**Affiliations:** 1 Department of Surgery, 4th Military Teaching Hospital, 5 Weigla Street, 50-981 Wroclaw, Poland; 2 Division of Anaesthesiological and Surgical Nursing, Department of Nursing and Obstetrics, Faculty of Health Science, Wroclaw Medical University, 5 Bartla Street, 51-618 Wroclaw, Poland; 3 Department of Pathomorphology, 4th Military Teaching Hospital, 5 Weigla Street, 50-981 Wroclaw, Poland; 4 Department of Clinical Pathology, Wroclaw Medical University, 213 Borowska Street, 50-556 Wroclaw, Poland; 5 Department of Internal Medicine, Occupational Diseases, Hypertension and Clinical Oncology, Wroclaw Medical University, 213 Borowska Street, 50-556 Wroclaw, Poland

**Keywords:** Nectin, Necl, cancer, colorectal, diagnostics

## Abstract

In 2020, colorectal cancer was the third most common type of cancer worldwide with a clearly visible increase in the number of cases each year. With relatively high mortality rates and an uncertain prognosis, colorectal cancer is a serious health problem. There is an urgent need to investigate its specific mechanism of carcinogenesis and progression in order to develop new strategies of action against this cancer. Nectins and Nectin-like molecules are cell adhesion molecules that take part in a plethora of essential processes in healthy tissues as well as mediating substantial actions for tumor initiation and evolution. Our understanding of their role and a viable application of this in anti-cancer therapy has rapidly improved in recent years. This review summarizes the current data on the role nectins and Nectin-like molecules play in colorectal cancer.

## 1. Introduction

Colorectal cancer (CRC) is a serious worldwide health problem with over 1.9 million new cases estimated in 2020 [1]. Globally, CRC is the third most commonly diagnosed cancer in both sexes combined [2]. It is estimated that CRC is the second most common cause of cancer-related mortality and accounts for approximately 930 thousand deaths around the world in 2020 [1,3]. Increase in incidence, altogether with predictive models for future years, prove that CRC is a growing burden for patients and national healthcare systems [4]. A significant shift toward the growing incidence among younger adults (<50 y.o.) makes those predictions even more worrisome [5]. The COVID-19 pandemic led to weighty health care delivery disruptions also in the matter of the prevention and treatment of patients with CRC [6,7]. The association between the risk of a more advanced stage of disease and the COVID-19 pandemic was reported in patients undergoing CRC when compared with pre-pandemic cases [8]. Limited access to health care and a CRC screening slowdown emphasized the importance of research in new diagnostic tools. Colorectal cancer is a nonhomogeneous group in which over 90% of cases are adenocarcinomas [9]. Long-term survival and cure rates have not improved significantly in recent years, especially in more advanced stages of the disease [9,10,11]. Carcinogenesis involves an accumulation of irreversible genetic mutations and epigenetic alterations. Within the same tumor, there are genetically distinguished cell populations coexisting. Epigenetic alterations vary from “hard-wired” and stable during cell division to transient and changing with every few divisions [12]. Epigenetic alterations, leading to developmental genes’ reactivation, normally silent in somatic tissues, have been observed in CRC specimens [13]. Not every genetical mutation and epigenetic alternation lead to specific phenotype change. Phenotypic plasticity has been described, also in CRC cells, showing that there is an ability to alter transcriptome without underlying genetic or epigenetic heritable mutation [14]. All the above mentioned sum up into a complex intra-tumor heterogeneity and are closely connected in cancer evolution. Clonal selection and evolution are at the base of treatment resistance [13]. Approximately 60% of all CRCs are sporadic–they develop without any known family history or any obvious genetic cancer syndrome (e.g., FAP or HNPCC) [15,16]. One-in-four CRCs has a hereditary component, as family and twin studies show. Yet, environmental factors play a role in carcinogenesis in those cases. Only 5% of CRC is attributed to well-characterized, high-penetrance syndromes [17].

The prognosis for patients with CRC is strongly determined by tumor invasiveness and the development of local or distant metastases. The presence of advanced locally or disseminated disease is a strong negative prognostic for a cure and long-term survival. Surgical treatment remains at the base of a curative approach [10]. Big differences in overall survival, even within same-stage groups, place the search for valuable prognostic factors in the spotlight of clinical research [18,19]. Among many others, cell surface molecules are being investigated for their clinical relevance (e.g., carcino-embryonic antigen). Cell adhesion molecules (CAMs) play a role in diagnostics and remain a field of interest in pathophysiology and the development of new treatment strategies in CRC [20,21,22,23]. With modern improvements we are on the verge of introducing a wide array of prognostic and predictive tools into clinical practice, which will help treatment decision making [24]. Advanced statistical tools can help with navigation through numerous biomarkers, combining individual and distinct features into more relevant groups–e.g., consensus molecular subtypes classification of CRC as a prognostic and predictive instrument for clinical decisions [25,26,27] Factors, once associated only with poor prognosis, lead researchers to new therapy development (e.g., BRAF, HER2) [28]. The new methods, such as circulating tumor DNA (ctDNA) allow more precise and sooner detection of the residual disease or relapse [29,30,31]. Nectins and nectin-like molecule seem to be worthy research candidates for future clinical implementation.

## 2. General Characteristics of Nectins and Nectin-like Molecules

Nectin and Nectin-like molecules (Necls) belong to immunoglobulin-like (Ig-like) transmembrane CAMs that play a significant role in cell-to-cell, calcium-independent adhesion formation, cell motility, proliferation, differentiation, polarization, and apoptosis. First discovered in 1986 as poliovirus receptor-related proteins (PRR proteins); then, after amino acid sequencing and further studies, they were identified as members of the immunoglobulin superfamily (IgSF). Finally, after discovering CAMs properties, a change in nomenclature was made: Nectins derived from latin “necto” i.e., “to bind” [32] (Table 1). Divided into four members of Nectins and five members of Necls, the superfamily gained attention due to their functions in immune response (e.g., TIGIT-related), being virion-entry cell receptors and tumor suppressors and oncoproteins [33]. Several experiments have shown lesser tumorogenic potential cells with positive expression of selected Nectins and Necls [34,35,36]. By contrast, the loss of expression of Nectins and Necls has been associated with a higher aggressiveness in several cancer cells e.g., Nectin-3 in pancreatic neuroendocrine tumor [37]. A specific mechanism of the mentioned tumor suppression is not well elucidated, whether contact inhibition is a main process is not clear. Studies about the evolution of Nectins and Necls have showed Nectin-like protein 5 to possess characteristics that are intermediate between these two groups. Moreover, sequence conservations of Nectins across species suggests evolutionary constraints due to the critical roles played by these CAMs [38]. Despite being diverse in terms of function, as well as phylogenetic distribution, members of the IgSF family share structural similarities, such as an extracellular domain consisting of three Ig-like loops, a transmembrane domain, and a short cytoplasmic tail [39]. On account of their ability to establish both, homophilic and heterophilic interactions with other CAMs (such as cadherins), Nectins perform a crucial role in the initiation of the formation of adherence junctions (AJs) [38].

### 2.1. Homophilic and Heterophicic Interactions

Nectins form transient and weak clusters of homo-cis dimers on the cell surface, which then trans-interacts with extracellular regions of opposing cell membrane Nectin molecules. It was demonstrated that Nectins are able to form both homophilic and heterophilic trans-interaction; however, heterophilic interactions are selective—for example, Nectin-1 interacts with both Nectin-3 and Nectin-4, but there is no interaction with Nectin-2 [40]. The selectiveness of heterophilic interactions is probably due to conserved charged residue localized in the center of the adhesive face of each Nectin. Therefore, only Nectins with opposed charged residues form heterophilic trans-interactions. Additionally, the transient and weak nature of homo-cis dimers can be, at least partially, explained by the repulsion of identical electrostatic charges [41].

Recruitment of E-cadherin to Nectin-based cell–cell adhesion sites and the subsequent activation of Cdc42 and Rac small G proteins enhances the formation of cadherin-based AJs through the reorganization of the actin cytoskeleton [42].

### 2.2. Nectin Spots

The C-terminus of the Nectins cytoplasmic domain determines their ability to interact with afadin and PAR3 [39]. Additionally, Nectins initiate afadin- and cadherin-independent cell adhesion apparatus that is not linked with the cytoskeleton. This type of apparatus is observed as dots or short lines under an immunofluorescence microscope and is named Nectin Spots [43]. 

The cytoplasmic tails of Necls differ from those of Nectins and interact with various scaffolding proteins, such as MAGUK or TCTEX1—a subunit of the dynein motor complex. Necls are ubiquitously expressed and have a greater variety of functions than Nectins [44]. 

### 2.3. Crosstalk between Cells and Extracellular Matrix

Many studies on MDCK cells have proven that Nectins and Necls promote crosstalk between cell–cell junctions and cell–extracellular matrix (ECM) junctions [42,44]. Extracellular regions of Nectins interact with integrins during the formation of AJs. Integrins are responsible for interactions with ECM proteins. The integrin-mediated cell–ECM junctions positively or negatively regulate the formation and stability of cell–cell junctions through protein tyrosine kinases associated with integrins (e.g., FAK, c-Src). Interaction between Nectins is responsible for integrin inactivation and, therefore, the stabilization of AJs [45]. The breakdown of AJs leads to the disruption of tight junctions (TJs) and desmosomes and the loss of epithelial cell integrity by affecting subsequently cell–ECM junctions. This process may lead to epithelial-to-mesenchymal cell transition (EMT). In cancerous cells, such increased motility is responsible for pathological implications, i.e., invasion—EMT and metastasing—cell migration [46,47,48].

### 2.4. Interactions with Growth Factors

Growth factors are responsible for cell proliferation regulation. In mature tissues, that process is transduced by a complex network of molecules. Interactions between Nectins, Necls, and growth factor receptors play a significant role in this network. For example, Nectins interact with the platelet derived growth factor (PDGF) receptor through the extracellular region and regulates the PDGF-induced activation of Akt and the inhibition of apoptosis in cooperation with afadin at PI3K activation. Experiments on Nectin-3 or afadin knockdown resulted in attenuated phosphorylation of Akt as well as decreased activity of PI3K enhanced by the PDGF receptor in NIH3T3 cell lines. Furthermore, embryoid bodies derived from afadin displayed an enormous number of apoptotic cells [45,49].

### 2.5. Tissue Distribution

Nectins differ in tissue distribution, where Nectin-4 normally occurs in placenta and embryonic tissues and Nectins-1 to -3 are commonly expressed in healthy adult tissues [50,51]. Many studies have shown an overexpression of Nectin-4 in numerous cancerous tumors, such as breast, pancreatic, urotheliary, ovarian, squamous cell carcinoma, and colorectal cancer [52,53] (Table 2). Necl-5 has a rather low expression in healthy adult organs but is overexpressed in many cancers. Necl-1 is specifically expressed in neural tissue whereas other members are ubiquitously expressed in tissues [39].

### 2.6. Nectins in Disease

Studies have shown that mutations in Nectin and Necls coding genes can lead to many diseases, such as cancer, ectodermal dysplasia, Alzheimer’s disease, stress-related mental disorders, viral infections, and cataracts [43]. Several Nectin-1 mutations cause disorders in ectodermally derived structures, such as lip/palate clefts, dental anomalies, and hypertrichosis—known as, e.g., Zlotogor–Ogur syndrome (CLPED1) or Margarita Islands Syndrome (CLPED2) [45]. Studies on the biology and pathology of Nectins and Necls has led to important innovations and clinical applications, such as FDA-approved therapy for treating locally advanced or metastatic urothelial carcinoma antibody–drug conjugate: enfortumab vendotin uses Nectin-4 as a target molecule for entering tumor cells [54,55]. 

## 3. Nectins and Necls in Cancer

### 3.1. Nectin-1

Nectin-1 is known as CD111, PRR-1, PVRL-1, and expressed under normal conditions on cells of the gastrointestinal tract, liver, gallbladder, female reproductive organs, and skin [50,51]. It is probably best studied as an entry receptor for the human herpes virus 1 (HSV-1) infection (Table 1). Nectin-1 has gained additional attention due to its interactions with CD96 (known also as Tactile) [56,57]. Abnormal expression of Nectin-1 can be observed in tumors of epithelial origin, such as cervical squamous cell carcinomas, cancer-associated fibroblasts of pancreatic ductal adenocarcinomas, CRCs and gastric cancers, or malignant transformations of keratinocytes. The observed expression varies from low to high, and the published data are not consistent in this matter [40] (Table 2). CD96/Nectin-1 interactions have not yet been fully explored, although studies have shown that cells with Nectin-1 expression have been more susceptible to NK-mediated cell toxicity compared to those with no expression [56]. It can be speculated that, if soluble ligands circulating in serum can competitively inhibit NK-cell activation, they can also induce the internalization and degradation of activating receptors on cell surfaces. Colon cancer might be a promising target for NK cell-based adoptive immunotherapy, and there is a need to investigate the role of Nectin-1 in further studies [58]. HSV-1 is a well-studied virus and was used as the first rationally designed replication-competent onco-lytic virus (RCOV). Moreover, a soluble variable domain of Nectin-1 was successfully used in an experiment as an “adaptor” for increasing the efficacy of an HSV-1 infection in CHO cells with no Nectin-1 expression. Based on these findings, cancerous cells, which display enhanced Nectin-1 availability, may serve as a receptor for HSV-1 viral oncolysis [59].

Nectin-1 tends to be a possible prognostic factor in the disease-free survival of patients with CRC. Research conducted by Tampakis et al. showed that Nectin-1 is strongly expressed in the cytoplasm of CRC cells in comparison to adjacent cells. Nectin-1 expression in colorectal cancer is associated with a significantly worse three-year progression-free survival, therefore, identifying a group of patients at high risk of an early recurrence of the disease [60]. These findings correlate with studies on pancreatic ductal adenocarcinoma patients. Yamada et al. reported that diffuse Nectin-1 expression in the cancer-associated fibroblasts of pancreatic ductal adenocarcinoma patients is associated with invasion, metastasis, and shorter survival [61]. It is important to note that colorectal endometriosis, which represents the most aggressive form of endometriosis, is characterized by both an increased expression of Nectin-1 and a decreased expression of Necl-2 [62].

### 3.2. Nectin-2

Nectin-2 (also known as CD112 and PVRL-2) is expressed in a vast number of adult tissues [51]. There is an overexpression of Nectin-2 in tumors of epithelial origin, such as squamous cell carcinomas and in adenocarcinomas (e.g., colorectal, esophageal, lung, pancreatic, and gallbladder cancer) (Table 2). Besides involvement in cell–cell adhesion, Nectin-2 interacts with immune cells by binding to different immune receptors, including CD226 (DNAM-1, DNAX accessory molecule-1), T-cell immunoreceptor Ig tyrosine (TIGIT)-based inhibition motif (ITIM) domain, and CD112R [63,64]. Coupling with receptors of CD8+ T-cells and NKs to modulate immune functioning by mediating immune-activating or inhibitory signaling in leukocytes. Necl-5 binds to the same immune receptors as Nectin-2, and it has been proven in MCA-induced tumors of Necl-5-deficient mice that Nectin-2 upregulation compensates Necl-5 absence in immune surveillance. In this study, Nagumo et al. pointed out that there is some sort of modulation of expression between Nectin-2, Necl-5, TIGIT, DNAM-1, and CD96 since they did not observe any difference in tumor growth in the Necl-5-deficient mice model compared to wild phenotypes [65]. Nectin-2 has been found to play an important role in the process of the NK cell-mediated killing of colon adenocarcinoma cells. A functional blockage of Nectin-2 with a specific antibody led to the significant inhibition of NK cell cytotoxicity to a colon cancer cell [58]. Platelet cloaking of circulating tumor cells and releasing transforming growth factor beta (TGFß) from platelets is an evasion method to hide from immune surveillance [66]. Cluxton et al. reported that cancer cells cloaked by platelets had a significantly reduced expression of Nectin-2 and Necl-5 on the tumor cell surface. Simultaneously, TGFß mediated the CD266 downregulation on NK cell surface. This suggests that the “immune decoy” mechanism is mediated by platelets. Platelet cloaking actively disrupts the CD226/CD96–Nectin-2/Necl-5 axis of circulating cancer cells recognition and plays a significant role in metastatic cascade [67].

An estimated 3–5% of patients with diagnosed CRC display HER2 amplification. In recent years, this has emerged as an actionable therapeutic target. There are treatments targeted at HER2 in clinical trials, e.g., with trastuzumab-deruxtecan ADC in metastatic CRC [68,69]. Nectin-2 has an affinity with the inhibitory immune receptor CD112R present in T-cells and NK cells [70]. Trastuzumab has limited action on cells with low HER2 expression. Antibody blockage of CD112R-enhanced NK cell cytokine production when NK cells were incubated with trastuzumab-coated breast cancer cells [71]. Thus, Nectin-2 and its receptors can be utilized to improve the therapeutic level of trastuzumab and can possibly be used in cases with lower HER2 expression. The complex and not yet fully explored TIGIT-CD96-CD112R-CD-226 axis is a promising target for a next generation of immunotherapy in cancer [57,64].

PVRIG binds with high affinity to Nectin-2 and suppresses T-cell function. This may lead to the presence of exhausted T-cell phenotypes in the tumor microenvironment (TME) that is unable to perform effective cytotoxicity. Whelan et al. found that PVRIG-Nectin-2 axis blockage enhanced cytokine production and the cytotoxic function of T-cells [72]. Immune-checkpoint inhibitors targeting the PVRIG-Nectin-2 axis in Nectin-2 positive cancers may be evaluated as potential drugs.

What is more, Nectin-2 is the target of post-translational modifications. Ubiquitination not only decreases Nectin-2 surface expression by targeting the protein for degradation but also by promotes Nectin-2 intracellular retention. Upon the inhibition of the ubiquitin pathway, increased Nectin2 surface expression renders tumor cells more efficiently recognized and lysed by NK cells by mediating the CD226-dependent co-stimulation of both NK and CD8+ T cells [73].

An interesting finding was described by Bekes et al. in a study on endothelial permeability and Nectin-2 downregulation. This study observed that patients with more advanced stages of ovarian cancer displayed an overexpression of Nectin-2 in cancer cells, a lower expression of Nectin-2 in peritoneal endothelium cells, and a significantly higher concentration of circulating vascular endothelial growth factor (VEGF). These findings advocate a thesis that the downregulation of Nectin-2 is responsible for increased endothelial permeability, which is VEGF driven [74]. These findings are in line with a study carried out by Russo et al. However, increased vascular coverage in the retina and spleen of Nectin-2-deficient mice was assumed to be caused rather by a defect in contact inhibition than other angiogenic factors (e.g., VEGF). Moreover, it has been demonstrated that Nectin-2 is involved in the homecoming process of T-cell entry into the spleen. Both T-cell entry routes into the spleen (through the red pulp and the marginal zone) were impaired in the Nectin-2-deficient model [75]. 

Karabulut et al. measured serum levels of Nectin-2 in 140 patients with diagnosed CRC. It was found that serum Nectin-2 levels in patients with both metastatic and non-metastatic CRC have a diagnostic value since there was a significant difference in baseline serum Nectin-2 levels between the whole group patients and the healthy control group (*p* < 0.001; for all, non-metastatic (stage II or III), and metastatic patients). Patients with elevated serum Nectin-2 concentrations had significantly less favorable progression-free survival rates compared with those with lower levels (median 5.8 v 9.1 months, respectively, *p* = 0.04). However, the results of this study did not show a statistically significant relationship between serum Nectin-2 concentration and overall survival or chemotherapy responsiveness [76].

This finding is in line with results obtained by researchers in different types of cancers. Liang et al. tested the significance of Nectin- 2 in pancreatic ductal adenocarcinomas. It has been shown that Nectin-2 expression is significantly correlated with clinical progression, as indicated by large tumor size and lymph node metastasis. Moreover, positive Nectin-2 expression is correlated with shorter survival in the cited study [77]. Nectin-2 also acts as an entry receptor for the Herpes simplex virus by interacting with viral glycoprotein D [40,78]. The role of Nectin-2 in colorectal cancer needs to be further elucidated.

### 3.3. Nectin-3

Nectin-3, also known as PVRL-3, PRR3, or CD113, plays a significant role in organ development (e.g., ocular, inner ear, and cerebral cortex development) and is widely expressed in healthy adult tissues (e.g., endocrine, gastrointestinal tissues, testis) (Table 1). Nectin-3 has the most versatile skill in terms of trans-interactions with other family members [39,40]. It is the only known Nectin that is expressed on the T-cell surface. Nectin-3 plays a significant role in T-lymphocytes extravasation. It trans-interacts with Nectin-2 on endothelial cells (localized near high endothelial venules) and facilitates the transendothelial migration of immune cells to secondary immune organs or surrounding tissues [79]. A similar mechanism may apply to malignant cells during disease dissemination [80]. Nectin-3 was identified as a mandatory C. difficile receptor for TcdB-mediated cytotoxicity as it is highly expressed in the colon. It may serve as a drug-target to prevent pseudomembranous colitis symptoms in C. difficile infections [81]. There are no specific studies on the clinical application of Nectin-3 expression in CRC. From the published data, we know that Nectin-3 expression is upregulated in lung adenocarcinomas, ovarian, and nasopharyngeal carcinomas (Table 2). Minawa et al. presented a study in which the membranous expression of Nectin-3 (normally absent in healthy lung tissues) was found to be associated with a poor prognosis for lung adenocarcinoma patients. Surprisingly patients who showed the membranous expression of Nectin-3 together with e-cadherin co-location had a better overall survival rate than in the case of the separate localization of both molecules within a tumor cell. In cases where Nectin-3 was expressed on a cell membrane with no expression of e-cadherin, the overall outcome was the worst of all patient groups. Thus, the membranous Nectin-3 that does not have a physiological function (the recruitment of E-cadherin) may contribute to increased tumor malignancy [82]. Zhao et al. established Nectin-3 and NRXN3 (both are members of CAMs) as downstream target genes of the zinc finger protein 582 (ZNF582). The hypermetylation of ZNF582 in nasopharyngeal carcinoma (NPC) is associated with higher migration, invasion, and metastasis. The restoration of ZNF582 led to the downregulation of Nectin-3 expression and the upregulation of NRXN3. Subsequent knock-out experiments and an in vivo model confirmed that Nectin-3 acts as an oncogene in NPC. This study also elucidated a new way of regulating Nectin-3 expression by ZNF582 [83]. Similarly, Xu et al. reported that Nectin-3 overexpression in ovarian cancer is associated with a worse overall survival rate. As an oncogene, Nectin-3 contributes to tumor progression in ovarian cancer. These results indicate that the expression of Nectin-3 upregulates the expression matrix metalloproteinases (MMPs 1 and 2) and leads to enhanced migration and invasion in OC cells by inducing ECM degradation in the area surrounding the tumor [84].

Meanwhile, Nectin-3 is downregulated in pancreatic adenocarcinoma and neuroendocrine tumors along with breast cancer. In both cases, the loss of Nectin-3 expression is associated with higher malignancy and a poorer prognosis. Martin et al. conducted a study in which induced overexpression of Nectin-3 in breast cancer cell lines reduced their motility and invasion properties. Established TJs, measured by trans epithelial resistance, also appeared to be “tighter”. Nectin-3 may be a key component in the formation of cell junctions and a putative suppressor molecule to the invasion and metastases of breast cancer cells [85]. 

Covering results were obtained by Hirabayashi et al. in their study on Nectin-3 expression in pancreatic endocrine tumors (PanNETs). Loss of Nectin-3 expression in PanNETs was associated with larger tumor size, a higher grade, lymphatic involvement, a higher Ki67 labeling index, an advanced pT-factor, lymph node metastasis, an advanced tumor stage, nonfunctioning tumors, and shorter disease-free survival. The authors presumed that the higher rate of cell proliferation may be promoted by the disruption of trans-interaction between Nectin-3 and Necl-5 [37,86]. In the case of pancreatic adenocarcinomas, reports on Nectin-3 expression are in line with previously cited studies, where diffuse expression in cancer cells was associated with a favorable prognosis [87].

More than 90% of microsatellite instability (MSI) colon cancers cells carry TGF-ß-receptor type II (TGFBR2) mutations. Lee et al. reported that the reconstitution of TGFBR2 in HCT116 colorectal cancer cells led to an increased sialylation of Nectin-3. However, overall synthesis and expression appeared to not be affected [88]. Available studies on Nectin-3 are not consistent and suggest that the role of Nectin-3 may be dependent on the histopathological type and location of the tumor. Further research to evaluate the role of Nectin-3 in CRC is needed. 

### 3.4. Nectin-4

This is probably the most extensively researched Nectin family member in cancer and disease in general, and in breast cancer in particular. Nectin-4, known also as PVRL-4 or PRR4, is strongly expressed in fetal tissues during development (Table 1). There is little or no expression in adult tissues, besides the placenta, throat, bladder, breast, stomach, esophagus, salivary gland (ducts), and skin (epidermis and sweat glands) [40,89,90]. The importance of Nectin-4 during embryogenesis can be illustrated with the example of ectodermal dysplasia syndactyly associated with missense mutations of the PVLR4 gene. Disturbed trans-interactions between Nectin-1 and Nectin-4 causing Rac1 pathway alteration and delayed AJs formation in mutant cells are responsible for phenotypic presentations of EDSS1 and CLEPED1 [45,91,92]. By contrast, it was observed that Nectin-4 is overexpressed in various types of tumors (e.g., colorectal, gastric, esophageal, urotherial, breast, ovarian, hepatocellular, non-small cell lung carcinoma, and renal papillary cell) [90,93,94,95,96,97,98,99] (Table 2). Its role as an oncogene is being investigated.

Nectin-4 has been identified as a biomarker of cancer stem cells (CSCs). CSCs have been recognized as the root of cancers’ initiation and the resistance of cancer cells to conventional chemo- and radiotherapies; hence, they are critical in the metastasis, recurrence, and thus, the disease-free survival of, e.g., colorectal cancer [100,101,102]. Siddharth et al. alleged that Nectin-4 is a CSC biomarker in the breast cancer model. Nectin-4 deletion inhibited the invasion of EMT/TME, a WNT-signaling cascade and an anchorage-independent growth [103].

Colon cancer cells exposed to 5-fluorouracil (5-FU, a core drug in CRC chemotherapy worldwide) increased endogenous Nectin-4 expression. The 5-FU sensitivity is inversely related to Nectin-4 expression in CRC cell line studies. Thus, it has been proposed that Nectin-4 is one of the factors related to 5-FU resistance. Nectin-4 coupling with afadin and subsequent cell growth induction through the Pi3k/Akt axis is a putative mechanism of resistance to 5-FU therapy in CRC cells. A combination of BCNU and resveratrol-induced apoptosis in 5-FU resistant colon cancer cells by decreasing Nectin-4 expression [104]. 

It has been experimentally demonstrated by Siddharth et al. that Nectin-4 is responsible for the induction of WNT/β-Catenin signaling via the Pi3k/Akt axis and promotes cancer stem cell proliferation as well as EMT and metastasing [103]. The self-renewal properties of circulating cancer cells induced by Nectin-4 expression are considered responsible for tumor aggressiveness and may play a role in EMT–TME transitions. A greater expression of Nectin-4 was observed in secondary tumors [89]. Nectin-4 has been associated with virtually every stage of tumor progression and the dissemination of disease. This statement is strongly supported by studies reporting Nectin-4 overexpression correlating with disease advancement and a worse prognosis in other cancer types [52,89,93,94,96,103,105,106,107,108,109]. Nectin-4 appears to increase with tumor grade (e.g., based on the tumor-nodes-metastasis classification). Measured overexpression was highest in relapsed tumors [89,107]. 

Nectin-4 has an impact on the cancer microenvironment. ADAM17 (a disintegrin and metalloproteinase 17)/TACE (a TNFα-converting enzyme) overexpressed in many cancers, including CRC, is capable of Nectin-4 cleavage under hypoxic conditions. As a result, the endodomain and ectodomain of Nectin-4 are released in a process called shedding [110]. 

Siddharth et al. has investigated the role of the soluble Nectin-4 ectodomain in the tumor microenvironment. It induces angiogenesis by direct interaction with endothelial integrin-ß4 via Src/PI3K/Akt/iNOS cascade [111]. Chatterjee et al. have provided experimental evidence that the Nectin-4 endodomain physically interacts with IMPORTIN-α (KARYOPHERIN-α2) and is translocated and accumulated in a nucleus, which activates DNA repair and enhances cell proliferation. In addition, nano formulated quinacrine (NQC) inhibits the action of both shedded Nectin-4 domains in in vitro assays [112]. There has been demonstrated an anti-angiogenic effect of curcumin and veliparib (a PARP inhibitor) through the deregulation of Nectin-4 in alleged DNA repair inhibition as well [113]. Taken together, ADAM-17 and Nectin-4 are putative targets for anticancer therapies [110,111,113,114,115].

Kedashiro et al. reported that Nectin-4 and p95-ErbB2 (one of the trastuzumab-resistant HER2 receptor splice variants) cooperatively activated the Hippo signaling-dependent SOX2 gene expression in the T47D serum-free suspension breast cancer cell line. It subsequently enhances cell proliferation in an anchorage-independent manner [116,117]. It has been outlined that the SOX2-β-catenin/Beclin1/autophagy signaling axis enhances chemoresistance, induces EMT, and is responsible for CSC properties in CRC cells [118]. Surviving away from ECM and the ability to proliferate in blood is one of the key features of malignant cells. Clusters of circulating tumor cells have been identified as well from the blood samples of CRC patients [99]. The mechanisms mentioned above and potential Nectin-4 involvement in CRC cell lines are yet to be elucidated.

Additionally, Nectin-4 tends to be involved in vasculogenic mimicry (VM)—the phenomenon of fluid conducting, microcirculatory channels lined by nonendothelial cells. VM channels are generated by pluripotent embryonic stem cells, highly invasive tumor cells, and the ECM in aggressive primary and metastatic tumors in order to provide a sufficient blood supply [119,120]. Zhang et al. reported a strong association between Nectin-4 mRNA expression, metastases, and an advanced disease stage in CRC patients. They also observed an association between VM, Nectin-4, and integrin ß-1 (ITGB1) presence. Additionally, Nectin-4, ITGB1 and VM were significantly associated with metastases and TNM stage, which features highly invasive CRCs. All the three parameters can be utilized as prognostic factors for CRC [97]. 

Sethy et al. indicated that Nectin-4 is also responsible for the promotion of lymphangiogenesis. Their study was conducted on specimens collected from breast cancer patients. Nectin-4 was found to have a predominant role in promotion of tumor-induced lymphangiogenesis by increasing lymphatic vessel density (LVD) and activating the chemokine axis (CXCR4/CXCL12) [107]. These mechanisms were also investigated in other cancer types [121]. Moreover, LVD in peritumoral tissues are associated with local recurrence and DSF in CRC [122]. Whether Nectin-4 induces lymphangiogenesis in CRC is a matter for future research. 

Furthermore, Nectin-4 serves as a measles virus (MV) entry receptor [123]. It gives an opportunity to utilize the oncolytic properties of MV in the treatment of Nectin-4-induced cancer. In the available literature, MV was reported to act as natural cancer killer cells for Burkitt’s lymphoma, Hodgkin’s disease, and squamous cell carcinoma [124]. Sugiyama et al. utilized the natural killing effect of MV in the treatment of induced breast cancer in mice. Their findings showed that recombinant MV specifically binds to Nectin-4 expressed on the surface of cells and additionally that it can reveal anti-cancer effects in several other malignancies, including CRC [125]. 

Antibody-based cancer therapies target specific antigens on cancer cells to deliver a highly cytotoxic payload to tumor sites by harnessing the exquisite specificity of monoclonal antibodies (mAb) as a delivery vehicle [90,126]. There are many (>200 at the time of submission) antibody–drug conjugates (ADCs) in clinical trials. Enfortumab vedotin (sold under brand name PADCEV) is an ADC consisting of a human anti-Nectin-4 antibody linked to the cytotoxic microtubule-disrupting agent monomethyl auristatin E (MMAE). It is an FDA-approved therapeutic for locally advanced or metastatic urothelial carcinoma. The activity of this ADC is under investigation in other cancers known to express Nectin-4 [127].

Likewise, bicycle toxin conjugates (BTCs) are a new class of anticancer agents that allow efficient and targeted delivery of toxin payloads into tumors. Bicyclic peptide is conjugated to a cytotoxic agent via a cleavable linker. The molecules of BTCs are much smaller than ADCs and exhibit better tissue distribution and tumor cell penetration [128]. The recently discovered BTC8009 is targeted against Nectin-4. Linked to MMAE, it has demonstrated at least equal efficacy as enfortumab vedotin in rodent models. In addition, the new BTC is also a putative anti-cancer drug against other Nectin-4-expressing cancers [129].

Thus, based on current knowledge, Nectin-4 expression could be used as a prognostic and potentially predictive factor in CRC. Molecule itself can be a putative target for the development of new therapeutics. Nectin-4 can be utilized for new target-specific probes to improve tumor visualization and metastases detection in single-photon emission computerized tomography (SPECT) [130].

### 3.5. Nectin-like Molecule 5

Nectin-like Molecule 5 (Necl-5), also known as polio-virus-receptor (PVR), Tage4, or CD155, is phylogenetically more closely related to Nectins than to other Nectin-like molecules (Table 1). It has probably diverged from Nectin-2 [38]. It trans-interacts with Nectin-3 on neighboring cells and also mediates cell–ECM junctions by binding to vitronectin. Necl-5 takes part in the contact inhibition mechanism. When there is no cell–cell contact, Necl-5 prevents Sprouty2 (Spry2) phosphorylation. After cells come into contact with each other, Necl-5 is downregulated by endocytosis following transient trans-interaction with Nectin-3. Unprotected Spry2 is tyrosine-phosphorylated by c-Src, which is activated by the PDGF receptor in response to PDGF, and subsequently inhibits PDGF-induced Ras signaling for cell proliferation [39,49]. Upregulation of Necl-5 in cancerous transformed cells that exceed the rate of internalization during cell–cell contact has been proven in studies to increase cell proliferation and hence tumor progression [44]. Necl-5 colocalizes with integrin on leading edges and takes part in growth factor-induced lamellipodia formation [49]. Necl-5 is upregulated through the Sonic hedgehog pathway as well as in Ras-mutated cells and allegedly induces cancer cells proliferation by inter alia shortening the G0/G1 phase [131]. 

Through interactions with CD226 and TIGIT, molecules present on leukocytes, Necl-5 together with Nectin-2 is a key regulator in cell-mediated immune response [57,132].

By binding to TIGIT, Necl-5 induces immunosuppression by the inhibition of NK cell and CD8+ T-cell cytotoxicity [133]. At the same time, Necl-5 has an affinity to the DNAM-1 molecule, which enhances immunological response by recognizing and killing tumors [134].

Necl-5 is known to be overexpressed on various types of malignant cells, including CRC [135,136] (Table 2). Zheng et al. evaluated Necl-5 in CRC cell lines under different conditions. They observed increased apoptosis, inhibited colony formation ability, and cell cycle arrest in the G1 phase in CRC cells after Necl-5 knockdown. In addition, the authors observed reduced expression of some cell-invasion-related molecules, such as FAK, Src, and MMP-2. Necl-5 knockdown inhibited Akt phosphorylation. Taken together, their findings support previous studies on that subject—Necl-5 attributes to tumor progression, invasion, and metastases in CRC cell lines and may be considered an anti-apoptotic factor in CRC [135]. Morimoto et al. indicated that Necl-5 augments the metastasis of cancer cells, including CRC, to the lungs. Necl-5 mAb blockage reduced secondary tumor formation in lungs by 60% in a mice model [46]. The authors suggested that cancer cells with high Necl-5 expression attach to CD226-expressing platelets. The mentioned process leads to platelet cloaking and enhances immune evasion of cancerous cells. Cell aggregates were arrested in pulmonary capillaries where extravasation and metastases formation take place [46,67].

There are many examples of the importance of Necl-5 in carcinogenesis, CRC progression, and dissemination. Necl-5 is also involved in immune surveillance and can act both as a tumor inducer and suppressor. Therefore, research on the application of Necl-5 in diagnostics and treatment strategies is recommended.

## 4. Conclusions and Future Perspectives

Nectins and Nectin-like molecules takes part in a plethora of essential processes in healthy tissues as well as mediating substantial actions in tumor initiation and evolution. Most of the crucial mechanisms of CRC carcinogenesis cannot be explained by a single factor, but nonetheless, Nectins and Nectin-like molecules are surely an important part of the entire picture. Our understanding of their role and their viable application in anti-cancer therapy is far from complete. Additional studies are needed to evaluate reliable Nectin and Necls expression patterns in CRC cells. Research into potential spatial distribution and sidedness are necessary to elucidate the role of Nectins in different CRC clinical presentations. There is a possible place for Nectins and Necls in a future standard biomarkers array. Nectin-4 tends to be the most achievable therapeutic target for novel CRC treatment options with completion of present and upcoming clinical trials. Based on the broad spectrum of available papers, Nectins and Necls may serve as diagnostic (e.g., PET probes), prognostic (e.g., OS and DFS), and predictive (selection of targeted-therapy regimens) tools in CRC. 

## Figures and Tables

**Table 1:** The Nectin nomenclature and viral cell-entry mediation examples.

Molecule	Other Names	Viral Cell-Entry Mediation
Nectin-1	CD111, PVRL-1, PRR-1, HVEC	HSV-1 ^1^, HSV-2 ^2^, PRV ^3^, BoHV-1 ^4^
Nectin-2	CD112, PRVL-2, PRR-2, HVEB	HSV-1 ^1^, HSV-2 ^1^, PRV ^3^, HHV-6B ^5^
Nectin-3	CD113, PRVL-3, PRR-3	-
Nectin-4	PVRL-4, PRR-4,	MeV ^6^
Nectin-like molecule 5	CD155, PVR, Tage4	PV ^7^

^1^ Herpes simplex virus 1, ^2^ herpes simplex virus 2, ^3^ pseudorabies virus, ^4^ bovine herpes virus-1, ^5^ human herpesvirus 6B, ^6^ measles virus, ^7^ poliovirus.

**Table 2:** Expression of Nectins and Necls in selected cancers.

Molecule	Colorectal Cancer	Breast Cancer	Urothelial Cancer
Nectin-1	↑ *	↑ *	- ***/↑ *
Nectin-2	↑ *	↑ *	NED ****
Nectin-3	NED ***	↑ */↓ **	NED ****
Nectin-4	↑ *	↑ */↓ **	↑ *
Nectin-like molecule 5	↑ *	↑ *	↑ *

↑ * = high expression, ↓ ** = low expression, - *** = standard expression, **** = not enough data.
